# Assessing European Egg Parasitoids as a Mean of Controlling the Invasive South American Tomato Pinworm *Tuta absoluta*

**DOI:** 10.1371/journal.pone.0048068

**Published:** 2012-10-29

**Authors:** Anaïs Chailleux, Nicolas Desneux, Julien Seguret, Hong Do Thi Khanh, Pascal Maignet, Elisabeth Tabone

**Affiliations:** 1 Institut Sophia Agrobiotech, French National Institute for Agricultural Research (INRA), Sophia-Antipolis, France; 2 Biotop, InVivo AgroSolutions, Valbonne, France; University of California, Berkeley, United States of America

## Abstract

The South American tomato pinworm (*Tuta absoluta*) has recently invaded Europe and is rapidly spreading in the Afro-Eurasian continent where it is becoming a major pest on tomato crops. Laboratory tests were undertaken to evaluate the potential of 29 European strains of *Trichogramma* parasitoids to control *T. absoluta*. In addition to the host itself, the host plant (tomato) was used during the laboratory tests in order to increase the chance of selecting the best parasitoid strains. *Trichogramma* females were placed with *T. absoluta* eggs on a tomato leaflet in tubes. We compared the parasitism of *T. absoluta* by the various *Trichogramma* species tested to the *Trichogramma* species currently commercially available for the pest control in Europe, i.e. *Trichogramma achaeae*. Thereafter, the more promising strains were tested on a larger scale, in mesocosm (i.e. cages in greenhouses) and in greenhouse compartments to evaluate efficiency of laboratory selected strains under cropping conditions. The most efficient strain from the laboratory screening trials did not perform as efficiently under the greenhouse conditions. We discuss differences in parasitism levels among species and strains and among the different scales tested in the experiments, as well as implications of these results for further screening for biocontrol agents.

## Introduction

Some of the most serious arthropod pests in agricultural landscapes are invasive species [1]–[3]. In the USA, for example, introduced arthropod pests have been estimated to cause losses of around $20 billion each year [4]. For most of the invasive pests, chemical pesticides are the dominant pest management method, contributing to additional production costs and negative side effects on non-target organisms and human health [4]–[7]. The tomato leafminer, *Tuta absoluta* Meyrick (Lepidoptera: Gelechiidae), is one of the most devastating tomato pests in South America [8]. This pest has recently invaded European and Mediterranean basin countries and in few years has become a major pest in both greenhouse and outdoor tomato crops [9], [10]. Biological control is a key component of integrated pest management programs in tomato greenhouses in Europe, but its sustainability is threatened due to the extensive use of pesticides to control *T. absoluta* and possible well known associated side effects of these products on biocontrol agents [6], [11], [12].

The possible use of parasitic wasps of the *Trichogramma* genus (Hymenoptera: Trichogrammatidae) as biological control agents of *T. absoluta* is currently considered in Europe because of the natural parasitism of *T. absoluta* by various *Trichogramma* species reported in South America and in Europe (e.g. in Italy) [13], [14] and the effective use of *Trichogramma pretiosum* Riley for inundative releases against *T. absoluta* in South American tomato crops [15]–[18]. *Trichogramma* parasitoids have already been successfully used in biological control of various lepidopteran agricultural pests [2], [16], [19]. They are easy to rear [20] and to release in open fields or protected crops. Every year, more than 32 million hectares are treated worldwide using *Trichogramma* spp. [21], [22], mostly through seasonal inundative releases [19], [23]. The success of the *Trichogramma* releases depends on the knowledge of the biological characteristics of the parasitoid species or strains used, and on their interactions with a specific host [24]–[26]. Selecting the *Trichogramma* species with the highest affinity for the target pest and for characteristics of the agro-ecosystem is crucial to the success of the biological control program [19], [27]. *Trichogramma achaeae* Nagaraja and Nagarkatti, is currently available in some European and North African countries for inundative biological control of *T. absoluta* [28]. *T. achaeae* was shown to be efficient in pilot experiments and lowered *T. absoluta* infestation levels in experimental and commercial tomato greenhouses [9], [29]. However, the efficiency of this parasitoid depends on the use of high quantities of parasitoids per release (as indicated by biocontrol companies; e.g. 250,000–1,000,000/ha per week [30]), the level of infestation by *T. absoluta*, and upon presence of other natural enemies on the crop. In addition, it is expensive to produce *T. achaeae* because the absence of diapause causes difficulty in storage and handling. Identifying a more efficient *Trichogramma* species would allow establishing an optimized economically-sound biological control program against *T. absoluta*.

The aim of the present study was to compare the efficiency of 29 *Trichogramma* strains in parasitizing *T. absoluta* eggs on tomatoes on three different scales: laboratory, mesocosm (cages in greenhouse) and in greenhouse compartments. We tested European *Trichogramma* strains from 11 different species. They were selected because (i) they were representative of the biodiversity of the European *Trichogramma* species, (ii) they were collected on hosts similar (size and/or ecology) to *T. absoluta* or on hosts present on tomato plants, and (iii) they showed characteristics (e.g. diapause, thelytoky, etc.) which make mass rearing easier i.e. cost-effective industrial production. A strain of *T. pretiosum* was also tested to compare other strains to one from the area of origin of *T. absoluta*. *T. achaeae* was chosen as the control species as it is already commercially available. Only the most promising strains under laboratory conditions were tested on a larger scale to assess the effectiveness of these selected *Trichogramma* strains under cropping conditions.

**Table 1:** Year of collection, initial host and host plant, country of origin and Thelythoky status (females produced from unfertilized) of the 29 *Trichogramma* strains studied.

Species	Geographic origin	Host plant (family)	Host moth or butterfly (family)	Thelytoky	Year of Collection
*T. achaeae*	Canaries Island	Tomato (Solanaceae)	*Chrysodeixis chalcites* (Noctuidae)	no	2010
*T. buesi*	Southern France	Cabbage (Brassicaceae)	*Mamestra brassicae* (Noctuidae)	no	2009
*T. cacoeciae 1*	Southern France	Carnation (Caryophyllaceae)	*Epichoristodes acerbella* (Tortricidae)	yes	2002
T. cacoeciae 2 *	Northern France	Vine (Vitaceae)	*Lobesia botrana* (Tortricidae)	yes	1989
*T. cordubensis 1*	Spain	Blackthorn (Rosaceae)	*Iphiclides podalirius* (Papilionidae)	yes	1999
*T. cordubensis 2*	Egypt a	Olive tree (Oleaceae)	*Palpita unionalis* (Pyralidae)	yes	2005
*T. cordubensis 3*	Portugal	–	*–* (Noctuidae)	yes	1994
*T. daumalae 1*	Southern France	Apple tree (Rosaceae)	*Cydia pomonella* (Tortricidae)	no	2009
*T. daumalae 2*	Bulgaria	Apple tree (Rosaceae)	*Cydia pomonella* (Tortricidae)	no	1998
*T. dendrolimi 1*	China a	–	*Palpita unionalis* (Pyralidae)	no	1998
*T. dendrolimli 2*	Italy	Vine (Vitaceae)	*Lobesia botrana* (Tortricidae)	no	1991
T. euproctidis 1 *	Switzerland	–	*–*	no	–
*T. euproctidis 2*	Egypt a	Sugar cane (Poaceae)	*Chilo sacchariphagus* (Crambidae)	no	1999
*T. euproctidis 3*	Southern France	Carnation (Caryophyllaceae)	*Olethreutes arcuella* (Tortricidae)	no	2002
T. evanescens 1 *	Northern France	Vine (Vitaceae)	*Lobesia botrana* (Tortricidae)	no	1990
*T. evanescens 2*	Northern France	Cauliflower (Brassicaceae)	*Argyrotaenia sphaleropa* (Tortricidae)	no	2002
*T. evanescens 3*	Turkey	Maize (Poaceae)	*Ostrinia nubilalis* (Crambidae)	no	2003
*T. evanescens 4*	Southern France	Geranium (Geraniaceae)	*Cacyreus marshalli* (Lycaenidae)	no	2005
T. evanescens 5 *	Southern France	Tomato (Solanaceae)	*Chrysodeixis chalcites* (Noctuidae)	yes	1982
*T. evanescens 6*	Germany	Maize (Poaceae)	*Ostrinia nubilalis* (Crambidae)	no	2009
*T. evanescens 7*	Southern France	Vine (Vitaceae)	*Lobesia botrana* (Tortricidae)	no	1990
*T. evanescens 8*	Southern France	Cabbage (Brassicaceae)	*Ephestia kuehniella* (Pyralidae)	no	1998
*T. evanescens 9*	Southern France	Cabbage (Brassicaceae)	*Ephestia kuehniella* (Pyralidae)	yes	1998
*T. evanescens 10*	Southern France	Tomato (Solanaceae)	*Ephestia kuehniella* (Pyralidae)	yes	2010
*T. oleae*	Yugoslavia	Olive tree (Oleaceae)	*Glyphodes unionalis* (Pyralidae)	yes	1972
*T. pretiosum*	Uruguay	Vine (Vitaceae)	*Argyrotaenia sphaleropa* (Tortricidae)	no	1995
*T. semblidis 1*	Southern France	Rice (Poaceae)	*Ephestia kuehniella* (Pyralidae)	no	1997
*T. semblidis 2*	Northern France	Cabbage (Brassicaceae)	*Plutella xylostella* (Plutellidae)	no	2002
*T. semblidis 3*	Southern France	Apple tree (Rosaceae)	*Cydia pomonella* (Tortricidae)	yes	2009

Asterisks indicate the strains for which diapause or quiescence capacity has been identified in our laboratory.

a strain not collected in Europe but species is present in Europe.

## Materials and Methods

### Biological Materials

The plants used in the experiments were five-week old tomato plants, *Solanum lycopersicum* L. cv. Marmande. They were grown in climatic chambers (24±1°C, HR: 65%, photoperiod 16L:8D) and a nutrient solution was applied daily. A colony of *T. absoluta* was set up using greenhouse-collected individuals in July 2009 at INRA, Alenya, France (initial number of individuals = 190). *T. absoluta* were reared in growth chambers (25±1°C, RH 70±10%, 16L:8D). Adults were kept in cages (55×75×80 cm), containing tomato plants. Adult moths were fed on honey placed on one wall inside the cages. The eggs used in the screening were between zero and 12 h old. Parasitoids used for the experiments originated from collections in various countries (Table 1). Dr. B. Pintureau from the French National Institute for Agricultural Research in Lyon (France) identified all species before the experiments. Stock colonies of parasitoids were reared on UV-irradiated eggs of a substitute host, *Ephestia kuehniella* (Zeller) (Lepidoptera: Pyralidae) (18±1°C, RH 70±10%, 12L:12D). Rearing was carried out in glass tubes (length: 4.5 cm; diameter: 0.7 cm) and the parasitoids were fed on honey. *Trichogramma* species were maintained for at least three generations at the temperature of 25°C on *E. kuehniella* eggs before experimentation. The parasitoids used in the screening were between 12 and 24 h old.

**Figure 1:**
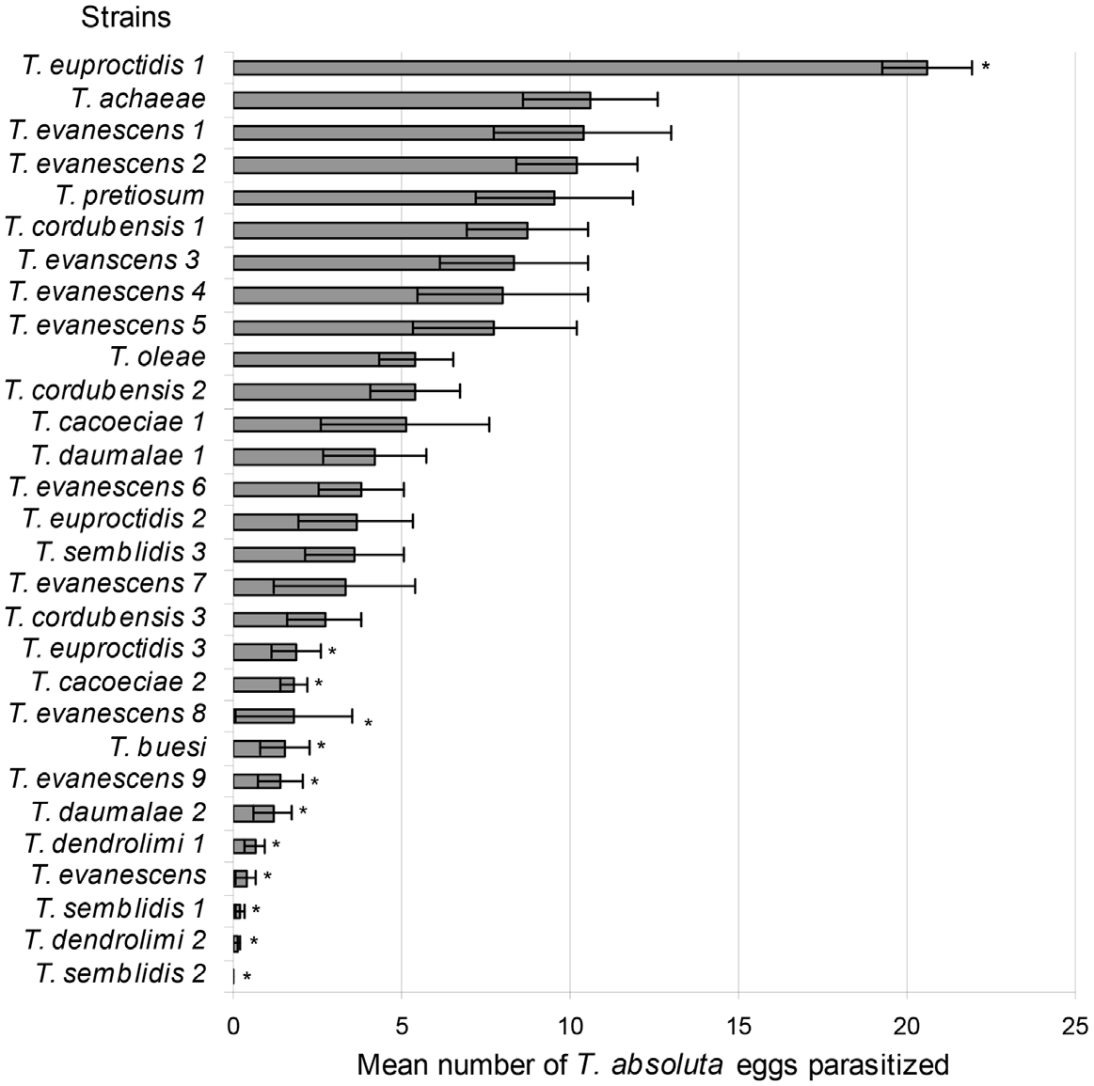
Parasitism of *Tuta absoluta* eggs under laboratory conditions. Mean (±SEM) number of parasitized *T. absoluta* eggs per *Trichogramma* strain in laboratory screening tubes on tomato leaflets**.** Strains with an asterisk are significantly different from *Trichogramma achaeae* at *P*<0.05 level (GLM analysis). One strain of *Trichogramma*, *Trichogramma euproctidis* 1, was significantly more efficient than *T. achaeae*.

### Laboratory Screening

The experiments were conducted in growth chambers at the temperature of 25°C (RH 70±10%, 16L:8D). Twenty-nine strains (among 11 species) were tested on *T. absoluta* eggs (designated as *strains* thereafter). Mated *Trichogramma* females were placed individually for 24 h with 30 *T. absoluta* eggs on a tomato leaflet (length: 7 cm ±0.5 SD) in a plastic tube sealed with a mesh to ensure ventilation (length: 14 cm × diameter: 4 cm), five drops of honey were deposited on the internal wall of the tube as food source for parasitoids. The 30 eggs per leaflet were obtained by releasing 15 *T. absoluta* (mixed males and females) on each leaflet in a tube overnight. Then, *T. absoluta* adults were removed and extra eggs were discarded (using a brush) to have 30 eggs per leaflet. The leaflet stem, sticking out of the tube, was planted into floral foam for watering. This design ensured that the leaflet stayed in good shape for the whole duration of the experiment. Between 10 and 15 replicates were conducted per parasitoid strain, and the replicates were carried out in a randomized order at different times. The tubes containing parasitized *T. absoluta* eggs were kept in the climatic chamber and maintained during five days. We counted the number of parasitized eggs (black eggs) and the number of aborted eggs (yellow non-hatched eggs). The proportion of females that parasitized at least one egg was also recorded.

**Figure 2:**
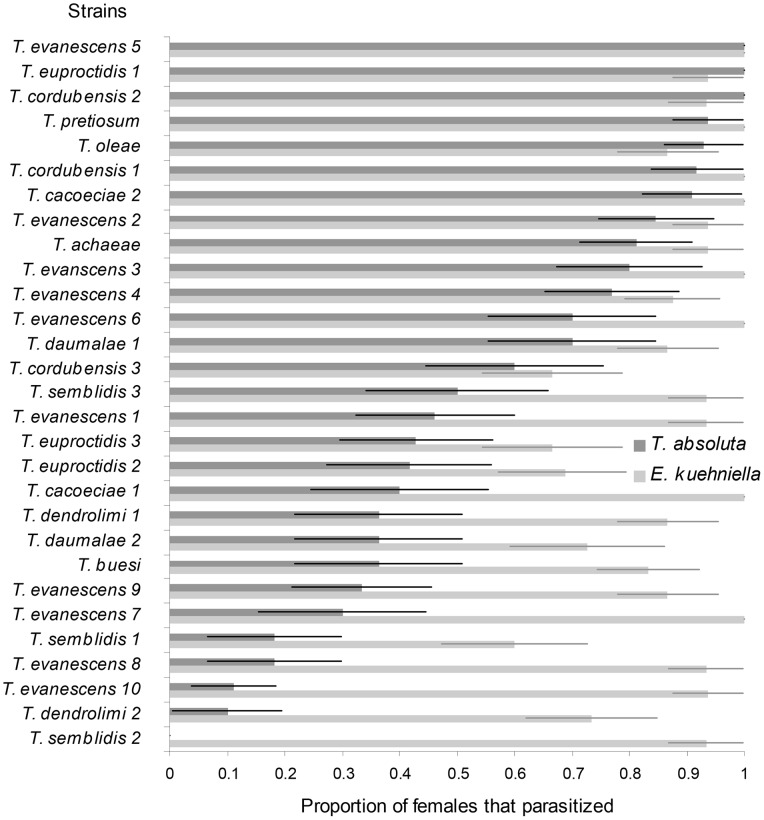
Acceptance of two hosts, *Tuta absoluta* and *Ephestia kuehniella*, by *Trichogramma* females. Data are presented as proportions (mean±SEM) of *Trichogramma* females that parasitized at least one egg on *T. absoluta* and on *E. kuehniella* in laboratory screening tubes.

**Figure 3:**
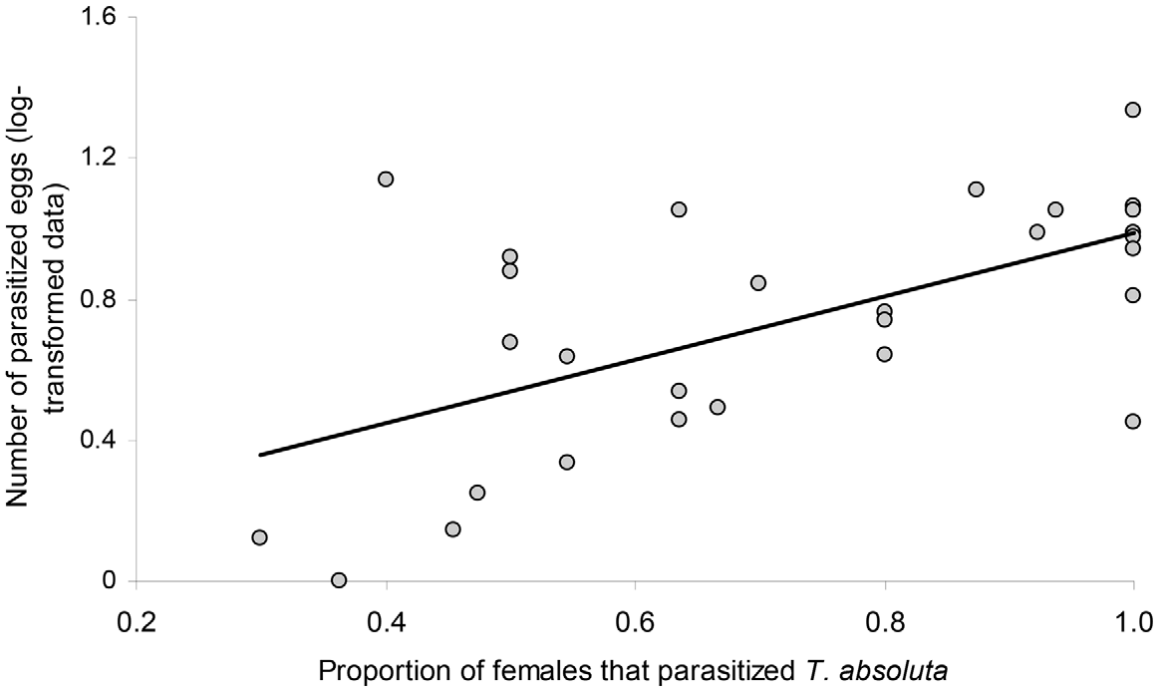
Preference-performance relationship. Data are presented as proportions of parasitoid females (for a given *Trichogramma* strain) attacking the host (*T. absoluta*) under laboratory conditions and the mean numbers of *T. absoluta* eggs parasitized (log-transformed data) (regression line: y = 0.9001×−0.0856).

In parallel to the tests using *T. absoluta* as host, experiments were also done using the rearing host *E. kuehniella* in order to compare biological characteristics of the various *Trichogramma* strains between the rearing and the targeted hosts. Mated parasitoid females were placed individually in glass tubes (containing honey as food) with 30–40 *E. kuehniella* eggs for 24 h in climatic chambers (25±1°C, RH 70±10%, 16L:8D). *E. kuehniella* eggs were glued on a strip of cardboard (3×10 mm) with 10% arabic gum. On each day of experiment, parasitoid strains were tested with the order of strains randomized (14–15 replicates per strain). The proportion of females that parasitized at least one *E. kuehniella* egg was recorded in the same way as tests using *T. absoluta* as tested host.

**Figure 4:**
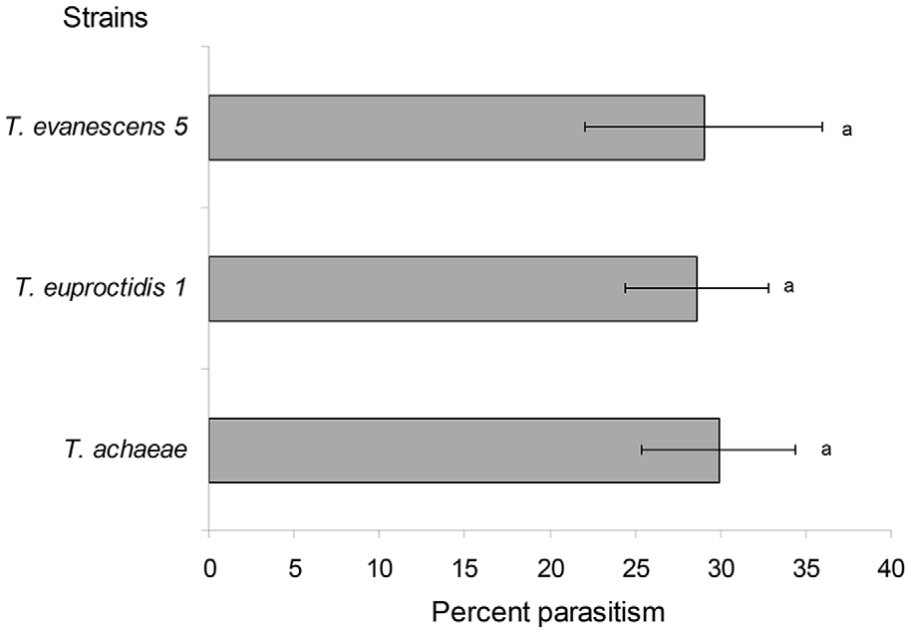
Parasitism of *Tuta absoluta* eggs in cages in greenhouse. Percentage (mean±SEM) of parasitized *T. absoluta* eggs per *Trichogramma* strain in cages in the greenhouse. Histograms bearing the same letter do not differ at *P*<0.05 (GLM analysis).

### Cage Experiments

The experiments were conducted in cages placed in a greenhouse located in Valbonne (French Riviera, France). Two species were compared to *T. achaeae* in cages: *Trichogramma euproctidis* 1 which showed the highest parasitism of *T. absoluta* eggs in laboratory trial (see *Results section*), and *Trichogramma evanescens* 5 which showed a similar level of parasitism as *T. achaeae* but that can be easily reared because of its thelytoky. The cages were placed in a glass greenhouse with semi-controlled temperatures, and the temperature was recorded in the cages with data-loggers during the whole experiment (min < mean temperature < max: 16.3°C <25.3°C <33.7°C; min < mean RH < max, 27.8% <70.8% <98.3%; natural ambient light: May-June 2011). Eight tomato plants (development stage from seven to 10 leaves) were put into cages (145×70×80 cm) covered by an insect-proof mesh. Twenty-five two-day old *T. absoluta* adults (mixed males and females) were released in the cages simultaneously with one of the *Trichogramma* strains. To release the parasitoids, small cardboard strips containing about 400 parasitized eggs of *E. kuehniella* from which parasitoids were just beginning to emerge, were placed in an open tube (drops of honey were provided as food source). The experiment lasted three days which is nearly the mean longevity of *Trichogramma* adults in tomato greenhouse (Chailleux A. and Desneux N., unpublished data). Then leaflets were collected (20–30 leaflets collected on upper, medium and lower part of the plant) until 100 eggs were found. Eggs were kept on the leaflets and placed into boxes in environmental cabinets (25°C, RH 70±10%, 16L:8D). Under these conditions, parasitized eggs become black in five days. The parasitized eggs were counted under a binocular microscope and the percentage of parasitism was calculated. We conducted seven to 12 randomized replicates for each *Trichogramma* strain (cage and order).

### Greenhouse Experiments

The most promising strain, *T. euproctidis* 1 (see *Results section*), was tested under greenhouse conditions and compared to *T. achaeae.* Experiments were conducted in two 60 m^2^ glass greenhouse compartments (min < mean temperature < max: 17.2°C <26.2°C <37.6°C; min < mean RH < max, 27.3% <71% <94.1%; natural ambient light: June-July 2011) located in Valbonne, French Riviera, France. Each greenhouse compartment contained three double rows of tomato plants under hydroponics cropping conditions. Forty adult moths (mixed males and females) were released simultaneously with 2,000 *Trichogramma* individuals. This situation mimicked a high level of infestation by the pest and a commercial release of *Trichogramma* for management of *T. absoluta* in tomato greenhouse (2,000 parasitoids for 25–100 m^2^). Parasitoids were released using the same method used in cages (i.e. on cardboard strips) at one central point of the greenhouse. A sample of 50 eggs was collected randomly in each compartment three days after the release and kept for incubation as described in the previous section (cage experiments). Six replicates were conduced for each *Trichogramma* strain, three in each compartment of the greenhouse.

### Statistical Analysis

All statistical analyses were performed using R software (R Development Core Team 2009) with the packages *multcomp* and *DTK*. For the laboratory experiments, the number of parasitized eggs (per female and per strain) were analysed using a generalized linear model based on Poisson distributed data with a log link function. Multiple comparisons were done using a Dunnett’s post-hoc test (comparison to the reference species *T. achaeae*). In addition, the effect of *Trichogramma* species, along with host plant family, host moth family and year of collection (for each strain) was also tested using a similar generalized linear model. Differences between the proportions of females parasitizing *T. absoluta* eggs on tomato compared to *E. kuehniella* (control host, i.e. rearing host) were tested using a generalized linear model designed for modelling binomial data with a logit link function. The assessment of preference-performance relationship [31] may be an important factor in choosing biological control agents [32]. Therefore, a linear regression analysis was used to assess the relationship between the mean number of parasitized eggs (log-transformed data) and the proportion of females that accepted *T. absoluta* eggs as host for each *Trichogramma* strain (i.e. proportion of females stinging [aborted eggs] or parasitizing [black eggs] at least one egg of *T. absoluta*). Finally, for the cages and greenhouse experiments, the percent parasitism was analyzed using a generalized linear model designed for modelling binomial data.

## Results

### Screening of 29 Strains on *T. absoluta*

The mean number of eggs parasitized in tubes varied significantly depending on the strain (F_28, 338_ = 10.907, *P*<0.001) (Fig. 1). The level of parasitism was significantly linked to *Trichogramma* species (F_10, 335_ = 8.296, *P*<0.001). The characteristics of the habitat of origin also had a significant effect on parasitism of *T. absoluta* by the parasitoids tested (host moth family: F_6, 329_ = 4.318, *P*<0.001, and host plant family: F_5,324_ = 7.328, *P*<0.001); strains originally collected from Noctuidae, Plutellidae and Crambidae, as hosts, and from Solanaceae, Oleaceae and Vitaceae, as host plants, parasitized the most *T. absoluta* eggs. In contrast, the year of collection was not significant (F_1,323_ = 0.146, *P* = 0.702). Only the strain *T. euproctidis* 1 was significantly more efficient than *T. achaeae* (Z = 3.379, *P* = 0.019). Another relevant strain was *T. evanescens* 5 because of its thelytoky, and because it showed a similar level of parasitism to *T. achaeae* (Z = −1.104, *P* = 0.999). Moreover, *T. pretiosum* was not significantly different from *T. achaeae* (Z = −0.428, *P* = 0.999) although it came from the same area as *T. absoluta*. Altogether sixteen strains were not significantly different from *T. achaeae*.

The proportion of females that parasitized the host varied across the 29 strains tested: in three strains all females parasitized *T. absoluta,* and in one strain (*Trichogramma semblidis* 2) none of the females parasitized the host. The proportion of females that parasitized *T. absoluta* on tomatoes was significantly different than on *E. kuehniella* (F_1, 56_ = 29.101, *P*<0.001) (Fig. 2). For a majority of the strains tested, the proportion of females that parasitized at least one egg was lower on *T. absoluta* on tomatoes than on *E. kuehniella* on cardboard. The linear regression analysis between the mean numbers of *T. absoluta* parasitized eggs and the proportion of females that accepted *T. absoluta* eggs, i.e. preference-performance assessment [31], [32], showed that strains parasitizing the most eggs also showed highest number of females accepting *T. absoluta* as host (R^2^ = 0.37, F_1, 27_ = 16.14, *P*<0.001) (Fig. 3).

### Cage Tests and Greenhouse Tests

Differences previously observed under laboratory conditions were no longer observed in cages (Fig. 4). The three strains (*T. achaeae*, *T. euproctidis* 1 and *T. evanescens* 5) showed similar efficiency against *T. absoluta* in cages; they all parasitized ∼30% of the eggs (F_2, 25_ = 0.019, *P* = 0.981). In greenhouse compartments, both *Trichogramma* strains tested (*T. achaeae* and *T. euproctidis* 1) were able to parasitize *T. absoluta*. *T. achaeae* showed the highest efficiency: 65.9±7.77% (mean ± SEM) as opposed to 19.4±2.73% (mean ± SEM) for *T. euproctidis*. In this case, the difference was significant (F _1, 11_ = 50.49, *P*<0.001).

## Discussion

Twenty-nine *Trichogramma* species-strains were tested under laboratory conditions and one strain of the *T. euproctidis* species (1) appeared promising (68.7% parasitism vs. 35.4% for parasitism for *T. achaeae* i.e. the species already commercialized in Europe and North Africa). Consequently this *T. euproctidis* strain was further tested on a larger scale i.e. in cages and greenhouses. However, the results of these later experiments did not corroborate the results obtained under laboratory conditions. Indeed the most efficient *Trichogramma* parasitoid against *T. absoluta* was still *T. achaeae*. Under cropping conditions, e.g. greenhouses, *T. euproctidis* was twofold less efficient against *T. absoluta* than *T. achaeae*. Our results showed that despite the fact that most *Trichogramma* strains did accept *T. absoluta* eggs on tomato leaflets, at least to some extent under laboratory conditions; the design was not sufficiently realistic to enable us to foresee their efficacy as a natural enemy of *T. absoluta* under real conditions.

The levels of parasitism of *T. absoluta* observed among the strains tested in the laboratory varied significantly. The low parasitism recorded for some strains may be attributed to two factors. First, in parasitoids, host specificity is mediated in part by host recognition and acceptance by the adult female parasitoid [25], [32], [33]. It has been shown that *Trichogramma* parasitoids prefer hosts with relatively big eggs [34] but the eggs of *T. absoluta* are, by comparison, three times smaller than the eggs of *E. kuehniella* used in the rearing. Furthermore, we found a positive relationship between the number of parasitized eggs and the proportion of females accepting *T. absoluta* as host, demonstrating that parasitism levels were directly linked to the willingness of females to attack the host (as demonstrated in other parasitoid systems [25], [32], [35], [36]). Second, low parasitism may result not only from rejection of *T. absoluta* eggs as host but rather from the poor capacity of some *Trichogramma* species/strains to cope with specific tomato plant characteristics. Various *Trichogramma* species have been reported to be highly susceptible to plant trichomes [37]–[41]. Although our study did not specifically assess the effect of trichomes on *Trichogramma* parasitoids, the experimental design was successful in identifying species having very little affinity for *T. absoluta*/tomato as host/host-plant complex.

Laboratory results also showed that the level of parasitism can differ greatly among strains of the same species. Variation within species of *Trichogramma* has already been encountered in other screenings [42]–[44]. Chassain and Bouletreau [45] studied the inter-strain variability of the main traits involved in *Trichogramma* parasitoid efficiency in host exploitation i.e. longevity, fecundity, progeny viability, progeny sex ratio and progeny allocation. They reported great differences among strains of the same species coming from different habitats, as well as between two different species coming from the same habitat. Consistent with these findings, our results showed that the characteristics of the original host and host plant of a given parasitoid strain, had an effect on its efficiency in parasitizing *T. absoluta* eggs, with strains originally collected from Solanaceae, Oleaceae and Vitaceae showing the best performance. Therefore, it is important to consider both initial host species and habitats when selecting strains of *Trichogramma* parasitoids for biological control programs.

The results from the cages/greenhouses did not match those from the laboratory; higher efficacy of *T. euproctidis* on *T. absoluta* disappeared when the scale of the experiments was increased. Differences between results under laboratory and greenhouse conditions may be due to both biotic and abiotic parameters. First, *Trichogramma* are known to be able to avoid plants bearing trichomes [46]. For some strains, females may have attacked *T. absoluta* eggs when constrained on tomato leaves in tubes in the laboratory but may have been able to avoid foraging on tomato leaves when released into cages or greenhouses. In addition, the oviposition pattern of hosts is a key factor for *Trichogramma* efficiency; *T. absoluta* does not lay egg masses but most of the time isolated eggs which thus increases the energy cost of foraging for hosts. Second, high temperatures, that are typical of greenhouses in summer, may impact differentially the various strains/species of *Trichogramma* [43], [47]. Moezipour *et al.* [48] indicated that there is a significant difference in the functional response of *Trichogramma brassicae* when tested at 20 or 30°C, and previous studies have shown that temperature and relative humidity can affect biological traits in *Trichogramma* spp. [43], [47], [49].

On the other hand, we could assume that differences between parasitoid efficiency recorded under laboratory and greenhouse conditions may result from the time that the different strains have spent under rearing conditions (year of collection). Efficiency under cropping conditions of one *Trichogramma* strain could be modified by the length of time spent under the rearing conditions in the laboratory, i.e. in tubes on alternative hosts such as *E. kuehniella* eggs, and at optimal temperature and humidity i.e. 25°C and 70% HR [50]. As the life cycle of *Trichogramma* parasitoids is usually short (egg to adult in about 11 days), adaptation to rearing conditions (humidity, temperature, mass-rearing host, confined environment, etc.) may occur. On the contrary, previous adaptations to given field conditions could disappear after a long spell of rearing under optimal laboratory conditions. Despite this, the collection year did not affect *Trichogramma* efficiency when assessed under laboratory conditions (no significant *year of collection* factor, *P* = 0.702). However it may impact parasitism only at a larger experimental scale (e.g. in greenhouse) where, for example, the foraging and dispersal capacities are key components of the parasitoid efficacy [33], [51]. Therefore, time spent in optimal rearing conditions may likely also partly explain some of the differences among strains tested in cages and greenhouse compartments.

We recorded the potential of various *Trichogramma* strains for biological control of *T. absoluta* in Europe. Nevertheless, our results did not identify that other *Trichogramma* strains showed better biocontrol traits than *T. achaeae*, i.e. higher fertility, high proportion of females/thelytoky and the capacity of diapause in cold storage in biocontrol company facilities. Further screening of *Trichogramma* parasitoids for potential management of *T. absoluta* would have to be based on the assessment of parasitoids collected on the targeted host in tomato crops under standard greenhouse cropping conditions in Europe. Doing this would increase the chances of assessing species that show greater likelihood of affinity within the cropping conditions. Colonies should be initiated with high number of field-collected individuals and new parasitoids should be added periodically. During laboratory screening, strains that showed very low parasitism levels were identified and removed; nonetheless, the efficacy of *Trichogramma* parasitoids under cropping conditions was not easily predictable from laboratory experiments. Studies could also be conducted directly in large cages (i.e. with multiple plants) in greenhouses when there are few strains to be tested. This way, all relevant criteria for strain selection could be taken into account and laboratory screening steps may be bypassed. Further studies would aim to (i) identify efficient parasitoids on *T. absoluta*, notably to prevent overuse of insecticides in tomato crops (and therefore ensure sustainability of current biological control and integrated pest management programs on this crop), and (ii) define new criteria that allow research and development programs at biocontrol companies to select accurately and quickly new *Trichogramma* strains (and more generally parasitoids) in the framework of biological control.
